# Positive feedback between retinoic acid and 2-phospho-L-ascorbic acid trisodium salt during somatic cell reprogramming

**DOI:** 10.1186/s13619-020-00057-1

**Published:** 2020-10-01

**Authors:** Mengdan Zhang, Qian Li, Tingting Yang, Fei Meng, Xiaowei Lai, Lining Liang, Changpeng Li, Hao Sun, Jiaqi Sun, Hui Zheng

**Affiliations:** 1 CAS Key Laboratory of Regenerative Biology, Guangzhou Institutes of Biomedicine and Health, Chinese Academy of Sciences, #190 Kaiyuan Ave., Science City, Guangzhou, 510530 Huangpu District China; 2 Bioland Laboratory (Guangzhou Regenerative Medicine and Health Guangdong Laboratory), Guangzhou, 510700 China; 3 Guangdong Provincial Key Laboratory of Stem Cell and Regenerative Medicine, Guangzhou, 510530 China; 4 University of Chinese Academy of Sciences, Beijing, 100049 China; 5 Guangzhou Medical University, Guangzhou, 511436 China; 6 Institutes for Stem Cell and Regeneration, Chinese Academy of Sciences, Beijing, 100101 China

**Keywords:** Reprogramming, L-ascorbic acid, Retinoic acid, Positive feedback, Mesenchymal-epithelial transition

## Abstract

Retinoic acid (RA) and 2-phospho-L-ascorbic acid trisodium salt (AscPNa) promote the reprogramming of mouse embryonic fibroblasts to induced pluripotent stem cells. In the current studies, the lower abilities of RA and AscPNa to promote reprogramming in the presence of each other suggested that they may share downstream pathways at least partially. The hypothesis was further supported by the RNA-seq analysis which demonstrated a high-level overlap between RA-activated and AscPNa activated genes during reprogramming. In addition, RA upregulated *Glut1/3*, facilitated the membrane transportation of dehydroascorbic acid, the oxidized form of L-ascorbic acid, and subsequently maintained intracellular L-ascorbic acid at higher level and for longer time. On the other hand, AscPNa facilitated the mesenchymal-epithelial transition during reprogramming, downregulated key mesenchymal transcriptional factors like *Zeb1* and *Twist1*, subsequently suppressed the expression of *Cyp26a1/b1* which mediates the metabolism of RA, and sustained the intracellular level of RA. Furthermore, the different abilities of RA and AscPNa to induce mesenchymal-epithelial transition, pluripotency, and neuronal differentiation explain their complex contribution to reprogramming when used individually or in combination. Therefore, the current studies identified a positive feedback between RA and AscPNa, or possibility between vitamin A and C, and further explored their contributions to reprogramming.

## Background

In 2006, Yamanaka and colleagues introduced four transcription factors into mouse embryonic fibroblasts (MEFs) and successfully generated induced pluripotent stem cells (iPSCs) (Takahashi and Yamanaka 2006). Then, the abilities of small-molecular compounds, including vitamins, to regulate reprogramming have attracted great attention (Esteban et al. 2010; Wang et al. 2011a).

Vitamin C (Vc) promotes reprogramming and increases the quality of generated iPSCs (Esteban et al. 2010; Esteban and Pei 2012). Mechanisms involving Vc-dependent H3K36 demethylases, *Nanog* and other pluripotency genes, and Ten-eleven translocation 1 (Tet1) have been reported (Wang et al. 2011b; Gao et al. 2013; Wu et al. 2014; Chen et al. 2013). Normally, 2-phospho-L-ascorbic acid trisodium salt (AscPNa) is used to provide L-ascorbic acid (Asc) during reprogramming with the help from phosphatase (Austria et al. 1997; Kameyama et al. 1996). Sodium-dependent vitamin C transporters (SVCTs) transport Asc into cells and maintain its intracellular concentration (Linster and Van Schaftingen E. Vitamin C. 2007). L-dehydroascorbic acid (DHAA), the oxidized form of Asc, is transported into cells by glucose transporters (GLUTs) and maintains intracellular concentration of Asc after being reduced (Linster and Van Schaftingen E. Vitamin C. 2007). The oxidation-reduction between Asc and DHAA and these membrane transporters, SVCTs and GLUTs, are critical for maintaining the balance of Asc across cell membrane (Wilson 2005).,

Vitamin A (Va) is normally obtained as *all-trans*-retinol, retinyl esters or β-carotene from diet (Mora et al. 2008). Retinoic acid (RA) is synthesized from retinol and activates the transcription of target genes after binding to its nuclear receptors. RA signaling pathway is reported to facilitate somatic cell reprogramming (Wang et al. 2011a; Yang et al. 2015). In addition, Deng and his colleagues generate iPSCs using chemical-defined medium which contains AM580 (agonist of RA receptor α) and TTNPB (a synthetic ligand of RA receptor) (Hou et al. 2013; Zhao et al. 2015; Masuda et al. 2013).

RA removes DNA methylation in embryonic stem cells (ESCs) by upregulating TET2 (Bar-El Dadon and Reifen 2017). In addition, both RA and AscPNa enhance the reprogramming to naïve pluripotency by modulating the levels and activities of TET proteins (Hore et al. 2016). Mesenchymal-epithelial transition (MET) is considered to be an essential process during early stage of fibroblast reprogramming (Li et al. 2010; Shu and Pei 2014). AscPNa promotes MET and facilitates reprogramming of MEFs (Chen et al. 2013). As a MET inducer, *Klf4* is also a downstream target of RA pathway (Li et al. 2017; Shi et al. 2012). Besides, RA is used as a potential chemo-therapeutic agent because of its important role in inhibiting epithelial-mesenchymal transition (EMT) (Cui et al. 2016; Guan et al. 2014). Thus, both RA and AscPNa regulate TET-mediated DNA demethylation and MET.

Therefore, an interaction between the downstream pathways of these two vitamins is possible, and the current studies were performed to confirm this hypothesis.

## Results

### AscPNa and RA impair the abilities of each other to promote reprogramming

To studies the functions of AscPNa and RA during reprogramming, three different kinds of media were used during reprogramming, including conventional mES medium, E8 medium, and N2B27 medium. mES medium contains approximately 15% fetal bovine serum (FBS) and is a traditional medium used for reprogramming (Esteban et al. 2010). The current E8 medium was modified from a previously reported chemical-defined medium, but without AscPNa, TGFβ or Nodal (Chen et al. 2011). N2B27 medium has been used to study the function of RA during reprogramming (Wang et al. 2011a), and B27 supplement without RA was used instead in the current studies.

**Fig. 1:**
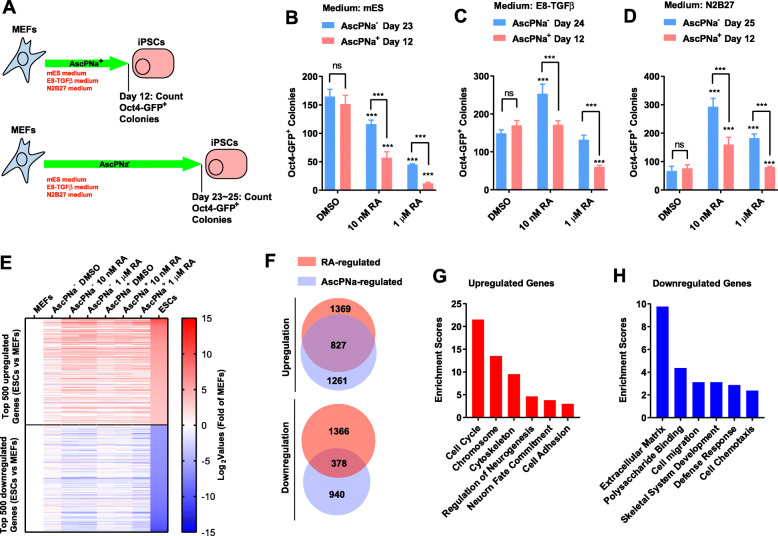
RA and AscPNa promoted reprogramming less in the presence of each other. **a-d** 10 nM RA or 1 μM RA were used during reprogramming of MEFs with or without 0.16 mM AscPNa (**a**). Three different media were used for reprogramming, conventional mES medium (**b**), N2B27 medium (**c**), and E8 medium (**d**). The Oct4-GFP^+^ colonies were counted on day 12 in the presence of AscPNa and on day 23–25 in the absence of AscPNa. By controlling the time points at which the Oct4-GFP^+^ colonies were counted, the numbers of Oct4-GFP^+^ colonies in “DMSO” groups without AscPNa were similar to those in “DMSO” groups with AscPNa. The comparisons were preformed between RA-treated groups and corresponding control (DMSO) groups or between indicated groups with two-way ANOVA (*n* ≥ 5, standard deviations were provided). (E-H) 10 nM or 1 μM RA were used during reprogramming of MEFs with N2B27 medium with or without 0.16 mM AscPNa. RNA-seq was performed on day 6. MEFs and ESCs were used as controls. Top 500 upregulated and downregulated genes were selected and their expression on day 6 during reprogramming was summarized in (**e**). Target genes of RA or AscPNa were selected (Log_2_ change over 1) and compared (**f**). The genes upregulated (**g**) and downregulated (**h**) by both RA and AscPNa were subjected to GO analysis

As indicated in Fig. 1a-d, the Oct4-GFP^+^ colonies were counted on day 12 in the presence of AscPNa. Actually, no Oct4-GFP^+^ colonies were identified on day 12 without AscPNa. Thus, AscPNa promoted reprogramming no matter what kind of medium was used (Fig. 1b-d).

The abilities of RA to promote reprogramming were concentration- and medium-dependent. Ten nM RA promoted reprogramming with N2B27 or E8 medium, while impaired reprogramming with mES medium (Fig. 1b-d). One μM RA had lower ability than 10 nM RA to promote reprogramming with N2B27 medium, but had higher ability to impair reprogramming with mES medium (Fig. 1b-d). Thus, the inhibitory effects of RA increased along with the increase of RA concentration, while the beneficial effects decreased.

By controlling the time points (day 23–25) at which the Oct4-GFP^+^ colonies were counted, the numbers of Oct4-GFP^+^ colonies in “DMSO” groups during reprograming without AscPNa were similar to those in “DMSO” groups during reprograming with AscPNa (Fig. 1b-d). Although the amounts of Oct4-GFP^+^ colonies were similar, the counting for reprogramming with AscPNa is much earlier (at least 11-day earlier) than that for reprogramming without AscPNa, which confirmed the abilities of AscPNa to promote reprogramming. In addition, when the amounts of Oct4-GFP^+^ colonies were similar in “DMSO” groups during two kinds of reprogramming, it is easier to identify the potential interaction between RA and AscPNa.

As indicated with N2B27 medium in Fig. 1d, the abilities of 10 nM or 1 μM RA to promote reprogramming were significantly impaired in the presence of AscPNa. In addition, although the amounts of Oct4-GFP^+^ colonies were similar in “DMSO” groups during two kinds of reprogramming, less Oct4-GFP^+^ colonies were observed in the presence of AscPNa than in the absence of AscPNa in “10 nM” or “1 μM RA” groups (Fig. 1d). Consistent results were obtained with mES or E8 medium in Fig. 1b-c. Therefore, RA and AscPNa have lower abilities to promote reprogramming in the presence of each other.

### RA and AscPNa share downstream target genes

There are several possible explanations for the observations in Fig. 1b-d. One is the cross-talk or overlap between the downstream pathways of RA and AscPNa. To confirm this, we performed RNA-seq on day 6 during the reprogramming with N2B27 medium. Top-500 upregulated and downregulated genes during reprogramming were selected based gene expression in MEFs and ESCs. The expression of these genes in the current RNA-seq results were summarized in Fig. 1e. Larger modulations of these genes in the presence of RA or AscPNa confirmed the abilities of RA and AscPNa to promote reprogramming.

The genes regulated by RA or AscPNa were then selected and analyzed. RA and AscPNa significantly upregulated 1369 and 1261 genes respectively, and 827 of them were upregulated by both molecules (Fig. 1f). This high-level overlap suggested that RA and AscPNa might share downstream pathways. Gene Oncology (GO) analysis indicated that these overlapping genes were enriched in categories related to cell cycle, neurogenesis, and cell adhesion (Fig. 1g).

The genes downregulated by RA and AscPNa were less overlapped. RA and AscPNa significantly downregulated 1366 and 940 genes respectively, and only 378 genes were downregulated by both molecules (Fig. 1f). These genes were enriched in categories related to extracellular matrix, cell migration, and cell chemotaxis (Fig. 1h).

### RA enhances the transportation of DHAA

One possible explanation for the overlap is that RA and AscPNa modulate the synthesis or degradation of each other. However, the genes which participate in the synthesis or degradation of Asc (based on Kyoto Encyclopedia of Genes and Genomes, KEGG, pathway 00053, ascorbate and aldarate metabolism) were not significantly modulated by RA during reprogramming (Table S1).

**Fig. 2:**
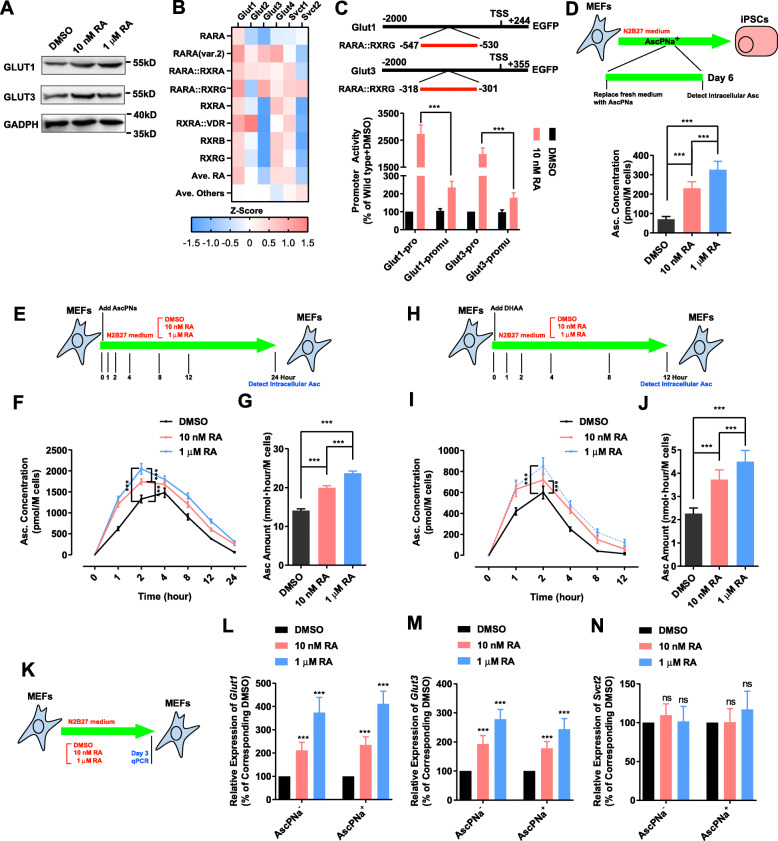
RA sustained the intracellular level of Asc. **a** The protein levels of *Glut1* and *Glut3* were determined by immunoblotting on day 6 during reprogramming with AscPNa. **b-c** The bind sites of RARA::RARG were identified on the promoters of *Glut1* and *Glut3* by Pscan software. Promoter assay confirmed the abilities of RA to affect the functions of the promoters of *Glut1* and *Glut3* in MEFs. **d** The intracellular level of Asc was determined on day 6 during reprogramming with N2B27 medium (24 h after replacing medium with fresh medium containing AscPNa). **e-j** MEFs were cultured with N2B27 medium containing 0.16 mM AscPNa or equivalent concentration of DHAA (E&H). Different concentrations of RA were also used. The intracellular levels of Asc was determined at different time points during the treatment with AscPNa (F&I) or DHAA (**f**), and the area under curve (intracellular levels of Asc multiple time length) were summarized in (**g**) and (j) respectively. **k-n** Different concentrations of RA were used to treat MEFs cultured in N2B27 medium in the presence or absence of 0.16 mM AscPNa (**k**). The expression of *Glut1* (**l**), *Glut3* (**m**), and *Svct2* (**n**) was determined by qPCR. The comparisons were preformed between RA-treated groups and corresponding control (DMSO) groups or between indicated groups with one-way ANOVA except with two-way ANOVA in (**c, f** & **j**). Experiments were repeated for at least five times (*n* ≥ 5). Standard deviations were provided

SVCTs and GLUTs which respectively transport Asc and DHAA into cells and maintain intracellular concentration of Asc (Linster and Van Schaftingen E. Vitamin C. 2007) were then analyzed. The expression of *Glut2*, *Glut4*, and *Svct1* were not detected in the current RNA-seq (Table S1). In addition, RA significantly upregulated the membrane transporters of DHAA, *Glut1* and *Glut3* (also known as *Slc2a1* and *Slc2a3*), but did not affect the expression of *Svct2* (also known as *Slc23a2*) (Table S1). Increase of the protein levels of *Glut1* and *Glut3* were observed on day 6 during reprogramming (Fig. 2a). Pscan software identified binding site of RARA::RARG on the promoters of *Glut1* and *Glut3* (Zambelli et al. 2009; Meneses et al. 2008; Lu et al. 2020), which was confirmed when the activities of their promoters of (both wild type and RARA::RARG binding site mutation) were determined in the presence and absence of RA (Fig. 2b-c). RA treatment increased the activities of the wild type promoters of *Glut1* and *Glut3,* but not the promoters with the binding sites of RARA::RARG mutated (Fig. 2b-c).

During reprogramming with N2B27 medium containing AscPNa, the intracellular level of Asc was determined on day 6 (24 h after replacing the medium with fresh medium). RA increased the intracellular Asc in a concentration-dependent manner (Fig. 2d).

The intracellular level of Asc was also determined in MEFs in the presence of AscPNa or DHAA (in N2B27 medium) (Fig. 2e-j). Since DHAA is less stable than AscPNa or Asc (Wilson 2002), the intracellular level of Asc was monitored for 12 h in the presence of DHAA, while 24 h in the presence of AscPNa. During the treatment of DHAA or AscPNa, the intracellular Asc increased at the early stage and gradually decreased to basal level at the late stage (Fig. 2e-j). RA treatment accelerated the initial increase of intracellular Asc at the early stage and impaired the decrease of intracellular Asc at the late stage (Fig. 2e-j). Thus, RA helps to sustain the intracellular level of Asc. In addition, when used in MEFs, RA significantly upregulated the expression of *Glut1* and *Glut3* but not *Svct2* (Fig. 2k-n). Therefore, RA maintains higher intracellular Asc by facilitating the DHAA transportation across cell membrane.

### AscPNa impairs the degradation of RA

**Fig. 3:**
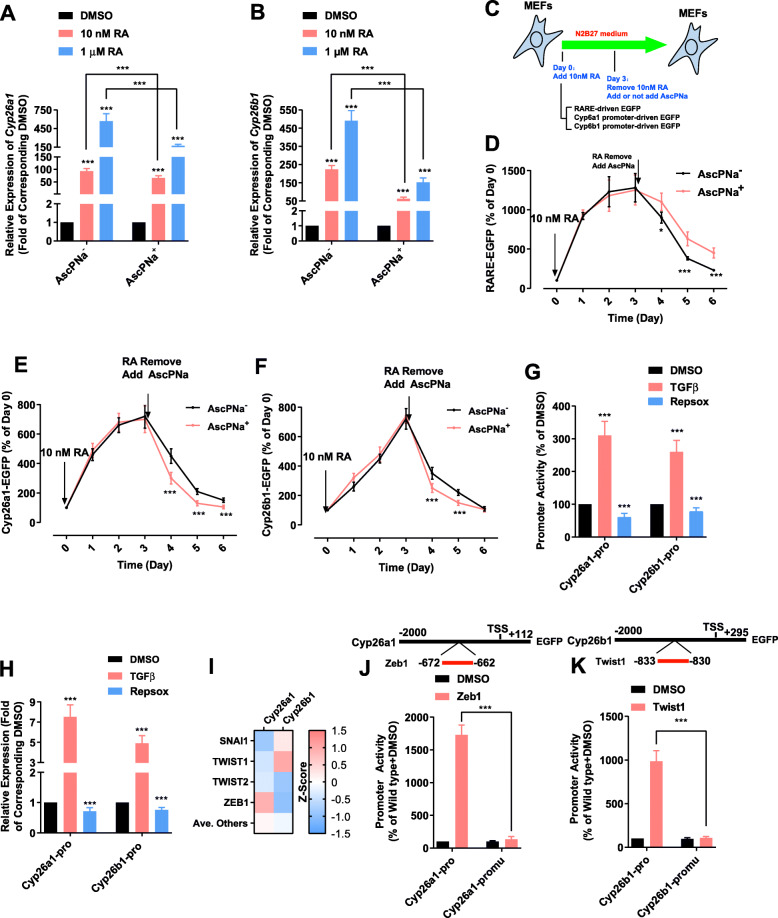
AscPNa impaired the degradation of RA. **a-b** Different concentrations of RA were used during reprogramming of MEFs with N2B27 medium in the presence or absence of 0.16 mM AscPNa. The expression of *Cyp26a1* (**a**) and *Cyp26b1* (**b**) was determined by qPCR. **c-f** EGFP reporter genes driven by different promoters were delivered to MEFs via lentivirus system (**c**). N2B27 medium was used 24 h after the delivery. 10 nM RA was included in the medium for the next three days. After the removal of RA, 0.16 mM AscPNa and PBS were used in two different groups. EGFP fluorescence was determined at indicated time points. RARE-containing mini promoter was used to indicate RA concentration in (**d**). Promoters of *Cyp26a1* (**e**) and *Cyp26b1* (**f**) were also used. **g-h** MEFs were cultured in N2B27 medium. Different concentrations of RA were used for three days. The activities of the promoters of *Cyp26a1* and *Cyp26b1* (**g**) and the expression of *Cyp26a1* and *Cyp26b1* (**h**) were determined by FACS and qPCR, respectively. **i-k** The bind sites of ZEB1 and TWIST1 were identified on the promoters of *Cyp26a1* and *Cyp26b1* by Pscan software, respectively. Promoter assay confirmed the abilities of ZEB1 and TWIST1 to affect the functions of the promoters of *Cyp26a1* and *Cyp26b1.* The comparisons were preformed between RA-treated groups and corresponding control (DMSO) groups or between indicated groups with one-way ANOVA except in (**d-f**). The comparisons in (**d-f, j-k**) were preformed between AscPNa^+^ and AscPNa^−^ groups or between pro or promu with two-way ANOVA. Experiments were repeated for at least five times (n ≥ 5). Standard deviations were provided

The ability of AscPNa to affect the intracellular RA was then determined. RA is normally degraded by CYP26A1 and CYP26B1 (Gottesman et al. 2001). The expression of these two genes was stimulated by RA in a concentration-dependent manner, which was suppressed at least partially in the presence of AscPNa (Fig. 3a-b).

Three RAREs and a mini-TK promoter were placed before *Egfp* reporter gene and used to determine the intracellular level of RA (Fig. 3c). The reporter was delivered into MEFs via a lentivirus system. MEF medium was replaced with N2B27 medium 12 h after an additional passage of MEFs. 10 nM RA treatment increased the EGFP fluorescence and the peak level was reached after three-day treatment (Fig. 3d). When RA was withdrawn on day 3, the EGFP fluorescence decreased gradually. In addition, adding AscPNa at the same time with RA withdrawal prevented the decrease of EGFP fluorescence. Thus, AscPNa sustains the intracellular level of RA.

The promoters of *Cyp26a1* and *Cyp26b1* were also placed before the *Egfp* reporter gene (Fig. 3c). RA treatment and RA withdrawal initiated the increase and decrease of *Egfp* expression, respectively (Fig. 3e-f). However, adding AscPNa at the same time with RA withdrawal (day 3) facilitated the decrease of *Egfp* expression. Therefore, AscPNa maintains the intracellular level of RA by suppressing *Cyp26a1/b1* and impairing RA degradation.

AscPNa treatment decreased the activities of *Cyp26a1* and *Cyp26b1* promoters, it is reasonable to suggest that several downstream transcriptional factors of AscPNa might bind to these two promoters. AscPNa-induced MET is a possible explanation. To confirm this, TGFβ and Repsox, a TGF pathway inhibitor, were then used to induce EMT and MET respectively in MEFs in the presence of 10 nM RA in N2B27 medium. TGFβ activated while Repsox inhibited the promoters of *Cyp26a1* and *Cyp26b1* (Fig. 3g)*.* In addition, TGFβ activated while Repsox inhibited the expression of *Cyp26a1* and *Cyp26b1* in MEFs (Fig. 3h)*.*

Binding sites of key mesenchymal transcriptional markers, like ZEB1 and TWIST1 were identified on the promoters of *Cyp26a1* and *Cyp26b1* by Pscan software (Zambelli et al. 2009; Hu et al. 2008; El Hokayem et al. 2018) (Fig. 3i). In addition, overexpression of *Zeb1* and *Twist1* increased the activity of wild type promoters of *Cyp26a1* and *Cyp26b*1 respectively, but not the promoters with the corresponding binding sites mutated. (Fig. 3j-k). Therefore, AscPNa suppresses the expression of *Cyp26a1* and *Cyp26b1* by inducing MET and suppressing these key mesenchymal transcriptional factors.

### RA has high ability to induce MET and pluripotency

The genes which were significantly regulated by RA but not by AscPNa were selected and analyzed. These genes were enriched in the categories related to extracellular matrix and cell motion (Fig. S1A-B). The RNA-seq results indicated that RA had high ability to induce MET (Fig. S1C).

As a MET inducer, *Klf4* can be upregulated by RA-related pathway (Li et al. 2017; Shi et al. 2012). To determine whether other key transcriptional factors in ESCs were also regulated by RA, top 500 upregulated genes during reprogramming were analyzed. Among these genes, *Nanog* and *Dppa5a* attracted great attention. To confirm the ability of RA to regulate the expression of these genes, RA was used to treat MEFs cultured in N2B27 medium for three days. RA increased the expression of *Klf4*, *Nanog*, and *Dppa5a* in a concentration-dependent manner (Fig. S1D-F).

### RA induces neuronal differentiation of PSCs

RA is reported to promote the neuronal differentiation of PSCs (Gudas and Wagner 2011). Thus, high concentration of RA may induce differentiation of iPSCs generated during reprogramming, and subsequently result in a lower reprogramming efficiency. Treatment with 1 μM RA preferentially upregulated genes related to embryonic morphogenesis, forebrain development and retinol metabolism (Fig. S2A-B).

To further confirm the abovementioned hypothesis, the expression of neuronal markers was analyzed in Table S1. The expression of three neural lineage markers, *Nes*, *Prom1* and *Ascl1*, were identified to be upregulated by RA during reprogramming (Table S1). When RA was used to treat MEFs cultured in N2B27 medium for three days, it increased the expression of *Nes*, *Prom1* and *Ascl1* in a concentration-dependent manner (Fig. S2C-E).

## Conclusions

**Fig. 4:**
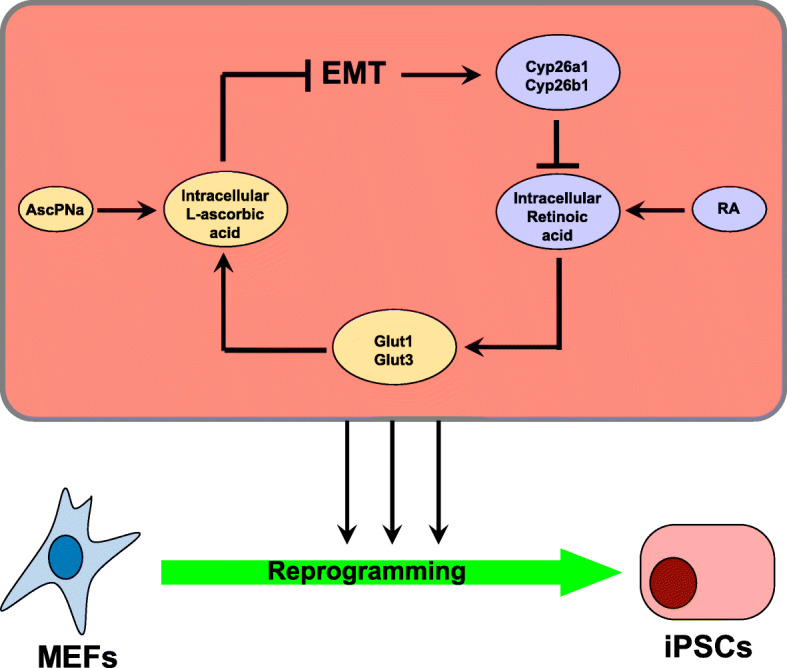
Schematic summary of the positive feedback between Va and Vc

In the current studies, positive feedback was identified between RA and AscPNa, during somatic reprogramming (Fig. 4). RA upregulates the expression of the membrane transporters of DHAA, *Glut1* and *Glut3*, maintains the intracellular level of Asc, and subsequently promotes reprogramming. AscPNa induces MET, suppresses the expression key mesenchymal transcriptional factors, resulting in the downregulation of *Cyp26a1* and *Cyp26b1*, and impairs the degradation of RA.

Although RA and AscPNa were not provided in N2B27 medium (B27 used in the current studies does not contain RA), the two vitamins were not eliminated totally during the current reprogramming. The retrovirus used to deliver four Yamanaka factors into MEFs was packaged in the Plat-E cells cultured in MEF medium (Table S1 and Materials and Methods). Since FBS is an important component of MEF medium, Va and Vc were provided to the cells via FBS during virus infection. The remaining Va and Vc in the cytosol of MEFs after changing MEF medium to N2B27 medium during reprogramming also prevented us to eliminate these vitamins totally.

When RA was used alone during the reprogramming with N2B27 medium, little Asc or DHAA remained in the medium. Thus, upregulation of *Glut1* and *Glut3* might not be able to sustain intracellular Asc. However, as reviewed by previous reports (Linster and Van Schaftingen E. Vitamin C. 2007; Wilson 2002), there are also mechanisms allowing the efflux of Asc or DHAA from cells. Thus, the membrane transporters for Asc or DHAA also function to maintain the balance between extracellular and intracellular Asc. Upregulation of *Glut1* and *Glut3* shifts the balance towards intracellular Asc and sustains intracellular Asc at higher level and for longer time.

The current positive feedback between RA and AscPNa provides additional understanding on these two vitamins (A and C) and reprogramming process. In addition, the RAREs on the promoters of *Gluts* and *Svcts* and the binding sites of mesenchymal markers on the promoters of *Cyp26a1* and *Cyp26b1* are consistent in different types of cells. In addition, some observations were not only observed during reprogramming but also confirmed during MEF culture. Thus, the current positive feedback may not be limited to somatic reprogramming and may be applicable in other biological processes.

## Methods

### Cell culture

All procedures related to animal were approved by the Institutional Review Board in Guangzhou Institutes of Biomedicine and Health. Mice were normally housed in groups of four with access to food and water ad libitum. After pregnancy, mice were housed individually with access to food and water ad libitum.

MEFs were isolated from 13.5-day mouse embryos carrying the Oct4-GFP transgenic allele (Szabo et al. 2002; Ying et al. 2002). Cells were cultured in MEF medium, which contains high glucose DMEM (Gibco), 10% FBS (Gibco), 1% GlutaMAX (Invitrogen), and 1% nonessential amino acids (NEAA, Invitrogen), for one passage before freezing in liquid nitrogen. MEFs were recovered from liquid nitrogen before use. MEFs were subjected to mycoplasma tests (MycoAlert™, Lonza) to ensure they were free of mycoplasma.

### iPSCs generation

The retrovirus plasmids pMX-Oct4, pMX-Klf4, pMX-Sox2, pMX-cMyc were delivered into Plat-E cells with by polyethyleneimine. MEF medium were used to culture Plat-E cells and viral suspensions were harvested at 48 h and 72 h after transfection.

The virus-containing medium was used to infect MEFs in 12-well plates (1.5 × 10^4^ cells/well) twice on day 0 and day 1. Corresponding medium was then used from day 2 to induce reprogramming and replaced with the fresh medium every other day. Reprogramming efficiency was evaluated by counting the number of Oct4-GFP^+^ colonies.

mES medium contains high glucose DMEM, 15% FBS, 1% GlutaMAX, 1% NEAA, 1% Sodium Pyruvate (Gibco), 0.1 mM β-mercaptoethanol, and 1000 U/ml leukemia inhibitory factor (LIF). N2B27 medium contains DMEM/F12 (1:1, Hyclone), 0.5% N2 supplement (Invitrogen), 1% B27 supplement (without Va, Invitrogen), 1% GlutaMAX, 0.1 mM β-mercaptoethanol, and 1000 U/ml LIF. E8 medium contains DMEM/F12, 19.4 mg/L insulin, 10.7 mg/L transferrin, and 14.7 ng/ml sodium selenium. Osmolality of all the media was adjusted to 340 mOsm at pH 7.4.

### qPCR and RNA-seq

RNA was extracted from cells with TRIzol reagent (Thermo Fisher). RNA-seq libraries were prepared for each RNA sample using the TruSeq RNA Sample Preparation Kit v2 (RS-122-2001, Illumina). The sequencing was performed with a NextSeq 500 High Output Kit v2 (75 cycles) (FC-404-1005, Illumina) following the manufacturer’s instructions. The depths of sequencing are 10 M pair-end reads of length 50NT. The raw data obtained from RNA-seq were tested for basic quality control with the FASTQC tool, and the filtered reads were pre-processed using the TRIMMOMATIC tool. The percentage of qualified reads was 97.37%. Reads were aligned to a transcriptome index generated from the Ensembl annotations (v67), using RSEM, bowtie2, and sequencing data using GC-content normalization (Risso et al. 2011).

After normalization, genes whose expression was significantly (above 1 or below − 1 when considering Log2 values) modulated by RA or AscPNa were used for GO analysis with DAVID 6.7 (https://david-d.ncifcrf.gov/) (Sherman and Lempicki 2009).

For qPCR, total RNA was extracted with Trizol. 2 μg total RNA was used to synthesize cDNA, which was then used to detect the expression of target genes by SYBR Green Master Mix ReverTra Ace qPCR-RT kit (TOYOBO). The expression of target genes was normalized to GAPDH. The primers for qPCR were listed in Table S2.

### Promoter activity assay

We used the plasmid pRlenti-EGFP as backbone to detect the promoter activity. We replaced the promoter of EGFP in pRlenti-EGFP with promoters of different genes, *Cyp26a1* (− 3000 ~ + 100 related to TSS), *Cyp26b1* (− 2000 ~ + 100), or three RA response elements (RAREs, CCGCTCGAGGGTGAACTTTCGGTGAACCCTACCC.

TCGAGTGAACTTTCGGTGAACCCTACCCTCGAGGGTAGGGTTCACCGAAAGTTCACTCGAC) plus a mini-TK promoter. Flow cytometry analysis was performed with Accuri C6 (BD Biosciences), the average EGFP fluorescence intensity of target cells were used to indicate the promoter activity.

### Intracellular Asc concentration

The intracellular Asc concentration was detected by ascorbic acid assay kit (fluorometric) (AB65346). The cells were digested with 0.25% trypsin and counted. Fluorescence was measured with a microplate reader at Ex/Em = 535/590 nm. Keep the whole process on ice and quick to avoid oxidation of Asc.

### Immunoblotting

Whole cell extracts were obtained with lysis buffer (50 mM Tris pH 8.0, 150 mM NaCl, 10% Glycerol, 0.5% NP40, 1 mM Phenylmethanesulfonyl fluoride and freshly added protease inhibitor cocktail). Approximately 100 μg of cell lysates were used for immunoblotting. Samples were loaded onto SDS-PAGE and transferred to PVDF membrane. The membrane was washed with TBST buffer and incubated with indicated antibodies (GLUT1, Abcam, ab115730; GLUT3, Abcam, ab191071; GADPH, Abcam, ab8245). Minichem Biosystem (SAGECREATION) were used to semi-quantify the target proteins.

### Statistical analysis

All experiments were repeated at least five times (*n* ≥ 5), with the exception of sequencing. The data were analyzed and compared using two-tailed t-test, one-way ANOVA, or two-way ANOVA with multiple comparisons in GraphPad Prism 7.0. Error bars and “n” represent the standard deviation (standard error if indicated) and the number of independent experiments, respectively. “*”, “**”, and “***” represent significant differences (*P* < 0.05, *P* < 0.01, and *P* < 0.001, respectively) from the indicated control groups.

## Supplementary information

**Additional file 1 Figure S1** RA had high ability to induce MET and pluripotency. **Figure S2**. RA promoted neuronal differentiation during reprogramming.

**Additional file 2.** Supplementary Table S1 RNA-seq results in the current studies.

**Additional file 3.** Supplementary Table S2 The primers used in the current studies.

## Data Availability

The RNA-seq datasets generated during the current study are available in Gene Expression Omnibus under accession numbers, GSE103791 and GSE142121 (https://www.ncbi.nlm.nih.gov/geo/query/acc.cgi?acc=GSE103791 and https://www.ncbi.nlm.nih.gov/geo/query/acc.cgi?acc=GSE142121).

## Abbreviations

AscPNa: 2-phospho-L-ascorbic acid trisodium salt
DHAA: L-dehydroascorbic acid
EMT: Epithelial-mesenchymal transition
ESCs: Embryonic stem cells
FBS: Fetal bovine serum
GLUTs: Glucose transporters
GO: Gene Oncology
iPSCs: Induced pluripotent stem cells
LIF: Leukemia inhibitory factor
MEFs: Mouse embryonic fibroblasts
MET: Mesenchymal-epithelial transition
NEAA: Nonessential amino acids
RA: Retinoic acid
SVCTs: sodium-dependent vitamin C transporters
Tet1: Ten-eleven translocation 1
Va: Vitamin A
Vc: Vitamin C
