# Supplementary material for: Positive feedback between retinoic acid and 2-phospho-L-ascorbic acid trisodium salt during somatic cell reprogramming
Source: Cell Regen. 2020 Oct 1;9:17. doi: 10.1186/s13619-020-00057-1 (PMC7527398; doi:10.1186/s13619-020-00057-1)
Supplement: Supplementary file 1 — Additional file 1 Figure S1 RA had high ability to induce MET and pluripotency. Figure S2. RA promoted neuronal differentiation during reprogramming. [file 13619_2020_57_MOESM1_ESM.pdf]

Positive feedback between retinoic acid and 2-phospho-L-ascorbic acid trisodium salt during somatic cell reprogramming

Mengdan Zhang[1,2,3,4,#], Qian Li[1,5,#], Tingting Yang[1,2,3,4], Fei Meng[1,2,3,4], Xiaowei Lai[1,2,3,4], Lining Liang[1,2,3], Changpeng Li[1,2,3], Hao Sun[1,2,3,4], Jiaqi Sun[2], Hui Zheng[1,2,3,4,6*]

[1] CAS Key Laboratory of Regenerative Biology, Guangzhou Institutes of Biomedicine and Health, Chinese Academy of Sciences, Guangzhou 510530, China

[2] Bioland Laboratory (Guangzhou Regenerative Medicine and Health Guangdong Laboratory), Guangzhou 510700, China;

[3] Guangdong Provincial Key Laboratory of Stem Cell and Regenerative Medicine, Guangzhou 510530, China;

[4] University of Chinese Academy of Sciences, Beijing 100049, China;

[5] Guangzhou Medical University, Guangzhou 511436, China;

[6] Institutes for Stem Cell and Regeneration, Chinese Academy of Sciences, Beijing, 100101, China.

[*]Correspondence to: Hui Zheng; Tel: 86-20-32015334; Fax: 86-20-32015231; Email: zheng_hui@gibh.ac.cn; #190 Kaiyuan Ave., Science City, Huangpu District, Guangzhou 510530, China.

Supplementary Information

Supplementary Information

Figure S1

RA had high ability to induce MET and pluripotency

(A-B) The genes which were regulated by RA but not by AscPNa were selected and subjected for GO analysis (upregulated genes in A, while downregulated genes in B). (C) The expression of epithelial and mesenchymal markers were used to quantify MET. (D-F) MEFs cultured in N2B27 medium were treated with different concentrations of RA for three days. The expression of *Dppa5a* (D), *Klf4* (E), and *Nanog* (F) was determined by qPCR. The comparisons were preformed between RA-treated and corresponding control (DMSO) groups with one-way ANOVA. Experiments were repeated for at least five times (n≥5). Standard deviations were provided.

Figure S1

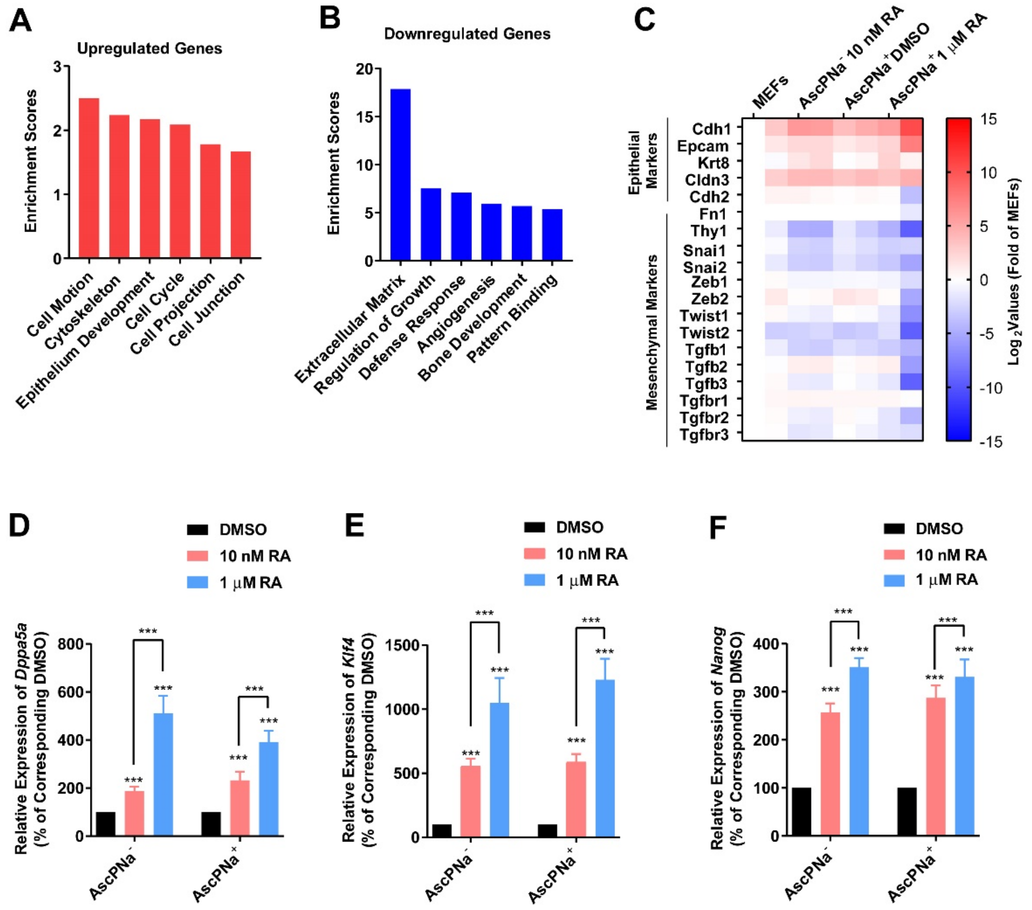

Figure S2.

RA promoted neuronal differentiation during reprogramming

(A-B) The genes which were regulated by 1 μM RA when compared to 10 nM RA were selected and subjected for GO analysis (upregulated genes in A, while downregulated genes in B).

(C-E) MEFs cultured in N2B27 medium were treated with different concentrations of RA for three days. The expression of *Ascl1* (C), *Prom1* (D), and *Nes* (E) was determined by qPCR. The comparisons were preformed between RA-treated and corresponding control (DMSO) groups with one-way ANOVA (n≥5).

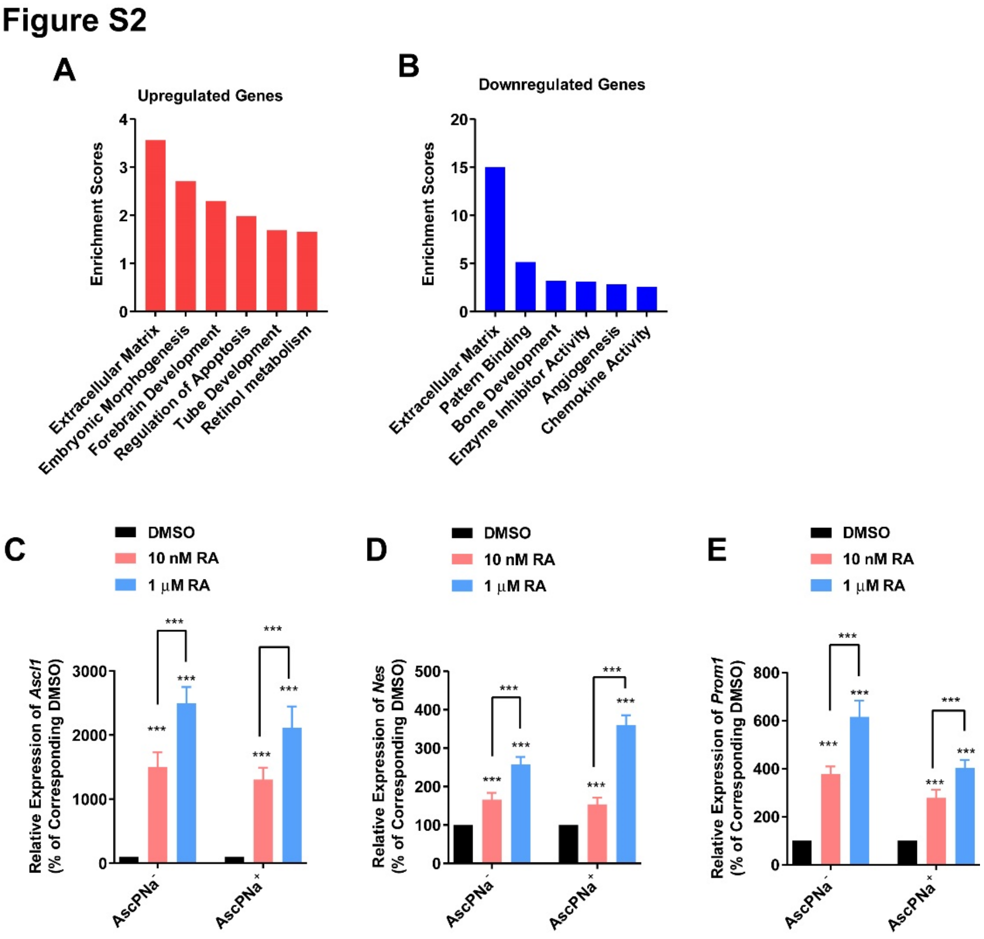

Supplementary Table S1

RNA-seq results in the current studies

10 nM or 1 μM RA were used during reprogramming of MEFs with or without 0.16 mM AscPNa. N2B27 medium was used. RNA-seq was performed on day 6. MEFs and ESCs were used as controls. The genes with no detectable expression were removed. The normalized expression was provided.

Supplementary Table S2

The primers used in the current studies

The primers used in the current studies for qPCR were listed.
